# Retrospective Analysis of Adult Patients Presenting to the Acute Care Setting Requesting Prescriptions

**DOI:** 10.5811/westjem.2021.6.52060

**Published:** 2021-09-24

**Authors:** Lisa Shepherd, Meagan Mucciaccio, Kristine VanAarsen

**Affiliations:** * Schulich School of Medicine and Dentistry, Western University, Department of Medicine, Division of Emergency Medicine, London, Ontario; † London Health Sciences Centre, Department of Emergency Medicine, London, Ontario

## Abstract

**Introduction:**

Patient visits to the emergency department (ED) or urgent care centre (UCC) for the sole purpose of requesting prescriptions are challenging for the patient, the physician, and the department. The primary objective of this study was to determine the characteristics of these patients, the nature of their requests, and the response to these requests. Our secondary objective was to determine the proportion of these medication requests that had street value.

**Methods:**

This was a retrospective, electronic chart review of all adult patients requesting a prescription from a two-site ED and/or an UCC in a medium-sized Canadian city between April 1, 2014–June 30, 2017. Recorded outcomes included patient demographic data and access to a family doctor, medication requested, whether or not a prescription was given, and ED length of stay. Medication street value was determined using a local police service listing.

**Results:**

A total of 2,265 prescriptions were requested by 1,495 patients. The patient median [interquartile range] age was 43 [32–54] years. A family doctor was documented by 55.4% (939/1,694) of patients. The two most commonly requested categories of medications were opioid analgesics 21.2% (481/2,265) and benzodiazepine anxiolytics 11.7% (266/2,265). Of patients requesting medication, 50.5% (755/1,495) requested medications without street value including some with potential to cause serious adverse health effects if discontinued. The requested prescription was received by 19.9% (298/1,495) of patients; 15.3% (173/1,134) returned for further prescription requests. The 90th percentile length of stay was 3.2 and 5.6 hours at the UCC and ED, respectively.

**Conclusion:**

Patients who presented to the ED or UCC sought medications with and without street value in almost equal measure. A more robust understanding of these patients and their requests illustrates why a ‘one-size-fits-all’ response to these requests is inappropriate and signals some fault lines within our local healthcare system.

## INTRODUCTION

Although they make up a small proportion of the overall visits,^1^ patients presenting to the emergency department (ED) or urgent care centre (UCC) for the sole purpose of requesting a prescription pose many problems: 1) for the patient, who may experience a long wait and possibly a mismatch between what they want and what the acute care service is willing to provide; 2) for the physician, who is in the business of episodic not comprehensive care and is diligently trying to avoid the misdirection of medications; and 3) for the department, which strives to conserve time and specialized resources that arguably should be directed toward patients with more urgent needs. Lacking in the literature is a closer examination of these patients who request prescriptions (PRP), which is needed to explore how they can be better supported to manage their health conditions.

Research to date has offered some insight into two groups of vulnerable ED patients with a close relation to PRPs. The first group consists of heavy utilization patients who make multiple visits to the ED.^2^ These patients have been shown to have not only unmet access needs but also significant economic and social forces driving their choices.^3^ The second group is patients who exhibit behaviors associated with prescription drug misuse.^4^,^5^ This is a complicated group that also intersects patients with pain and addiction issues.^6^ Requesting a prescription refill is one behavior that has been identified with prescription drug misuse.^4^,^5^ Patients in the ED who request prescriptions, make multiple visits, and exhibit prescription misuse behaviors are all subgroups of the very heterogeneous ‘non-urgent’ patient group for which a more robust literature exists.^7^–^9^ However, use of the ED for any type of non-urgent care remains controversial. Whether or not these visits contribute to ED crowding, increased costs, and deprivation of continuity of care remains unresolved.^10^–^12^

Obtaining prescriptions and navigating medical appointments are part of self-management of health conditions, which does not occur in isolation but rather in the context of patients’ physical, social, and family environment.^13^ Yet, before being able to consider a larger social determinants of health approach to these patients, we need to first understand PRPs and their requests. With this understanding, we may be better positioned to serve these patients and to support physician decision-making surrounding their care.

The primary objective of this study was to determine the characteristics of patients who present to the ED or UCC requesting a prescription, the nature of these requests, and the resulting action taken by the attending physician. The secondary objective was to determine the proportion of medication requests that have potential street value and the subsequent responses to these requests.

## METHODS

### Study design, setting and population

We conducted a retrospective analysis of electronic health record data^14^ between April 1, 2014–June 30, 2017. To capture the maximum number of patients, we used both the presenting complaint and discharge diagnosis of ‘issue of repeat prescription.’ The presenting complaint code was searched using the Canadian Emergency Department Diagnosis Shortlist,^15^ and the discharge diagnosis code was searched using the International Classification of Disease, 10^th^ Revision (ICD-10). We combined these lists to create our database. Any patient 18 years of age or older who attended either the ED or the UCC was included in the study. The study was approved by the Health Science Research and Ethics Board at Western University (no. 109752) and adhered to the STROBE (Strengthening the Reporting of Observational Studies in Epidemiology) guidelines for reporting observational studies.^16^

London Health Sciences Centre is a multisite, 1168-bed, quaternary hospital that serves an urban population of approximately 400,000. The two adult EDs at this hospital have a combined annual census of 165,000. The UCC is located between the two EDs geographically and sees 48,000 patients annually; it is open 365 days per year but closes in the evenings. A common pool of emergency physicians staffs all departments. At the time of this study, both EDs and the UCC site used a hybrid health record model, with physician notes recorded on a paper chart and all other data recorded electronically. Only the electronic data was accessed and collected for this study.

**Population Health Research Capsule**

What do we already know about this issue?

*Patients presenting to acute care settings for a prescription request are an underexplored group that present challenges for themselves and emergency care providers.*

What was the research question?

*What are the characteristics of patients who present for prescription refill, their requests, and the response to these requests?*

What was the major finding of the study?

*Patients sought medications with and without street value in almost equal measure and received prescriptions 20% of the time.*

How does this improve population health?

*A ‘one-size-fits-all’ strategy, such as diversion, is insufficiently nuanced to be adequate. These patients expose fault lines within local healthcare systems.*

### Outcomes

Trained research personnel recorded baseline patient demographics including age, gender, and whether or not a family doctor was identified. Repeat visits were checked amongst all three sites. We logged the day of week of presentation, wait time, length of stay (LOS), and whether the patient left prior to being assessed by a physician. In keeping with provincial reporting metrics, ED wait times and LOS were calculated as 90^th^ percentiles, which represent the maximum length of time in which 9 of 10 patients waited to be seen by a physician or completed their ED visit. We excluded patients who left prior to being assessed by a physician from wait time and LOS calculations. Specific medications requested were identified in the nursing triage note. Prescriptions issued at discharge were part of the electronic record and were documented and later categorized.

The separation of analgesics into opioid and non-opioid was accomplished using the triage nurse record of patient request. If the patient requested a medication by name, then their request was coded as opioid or non-opioid analgesic, respectively. If the patient requested ‘pain meds,’ then these were recorded as non-opioid analgesic to avoid overestimating opioids. Whether or not a medication had value on the street was determined using the London Police Service 2017 Street Drug Index, a report maintained and updated by the local police department, which lists medications and their expected monetary value when sold on the street.

### Data Analysis

Data were tested for normality and analyzed using descriptive statistics; they were summarized as mean (+-SD), median [IQR], or percentage as appropriate. Differences between groups were tested using Mann-Whitney U analysis. We defined *P* <0.05 as the level of statistical difference.

## RESULTS

**Figure 1:**
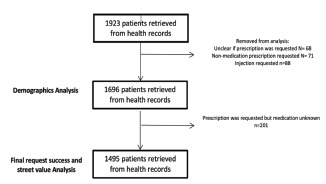
Patient health record selection process for the study.

A total of 1,923 cases met the inclusion criteria over the 39-month study period. We removed cases (n = 227) if it was unclear whether a prescription had been requested, or a non-medication prescription (ie, splint) or injection (ie, tetanus immunization) was requested (Figure 1).

### The Patient

The patient median age was 43 years [32–54] with 57.9% being male (Table 1). A family doctor was documented by 55.4% of patients. The EDs were chosen as the site of care by 38.6% (655/1,696) of patients while the UCC was chosen by 61.4% (1041/1,696). No significant difference was found for presentation by day of week. Some patients chose to leave before being seen by a physician, 24.1% (158/655) from the EDs and 8.1% (85/1,041) from the UCC. Of patients requesting a repeat prescription in the study time frame, 15.3% (173/1,134) had greater than one visit to either the EDs or UCC (range 1–26). Repeat presentations to the EDs only were seen in 22% (38/173), to the UCC only in 46.2% (80/173), and between the two facilities 31.8% (55/173). We compared demographic data for patients requesting at least one medication with street value to data for all patients.

### The Request

An additional 201 patients were removed from the remaining analyses due to an unknown medication being requested. A total of 2,265 prescriptions were requested by 1,495 patients with the median number of medications requested during the visit being 1 [1–2] with a range of 1–8. The most commonly requested medications were opioid analgesics, benzodiazepine anxiolytics, non-opioid analgesics, antidepressants, antipsychotics, and amphetamines (Table 2), with 39.1% (886/2,265) having street value. Of the patients presenting, 49.5% (740/1,495) made a request for at least one prescription of street value. Patients presenting with more than one visit had a similar profile of medication requests with 45.8% (362/791) having street value. Of these 173 multivisit patients, 57.8% (100/173) requested at least one prescription of street value.

### The Response

The 90^th^ percentile wait time for seeing a physician was 2.8 and 4.9 hours at UCC and EDs, respectively. The 90^th^ percentile LOS was 3.2 and 5.6 hours at UCC and Eds, respectively. The median time spent receiving care (ED LOS minus wait time) was 17 minutes [10–30] for patients who received their requested prescriptions and 20 minutes [11–36] for those who did not (P = 0.012). A total of 298 of 1,495 of patients (19.9%) received their requested prescription, with 7.9% (118/1,495) of patients receiving at least one prescription of street value. For all prescriptions requested, 27.2% (615/2,265) were written and 7.2% (162/2,265) had potential street value (shown in Table 2).

## DISCUSSION

To our knowledge, this is the first study to explore patients who present to acute care departments requesting prescriptions. Our study showed that approximately half of the patients presenting to an acute care department for the sole purpose of requesting a prescription asked for at least one medication that had value on the street. The other half requested a diverse array of medications without street value. Appreciation of this duality is important as we work to understand what drives these patients to seek care in acute care settings and subsequently strategize best care for these patients. This work adds to the body of literature by characterizing a previously poorly understood patient group; it adds to our local public health information by shining a light on some fault lines in the provision of healthcare within the community.

The median age of patients in our study (43 years) aligns well with previous studies examining non-urgent visits to the ED.^8^,^9^,^18^ Patients experienced long waits to be seen by a physician at both the UCC (2.8 hours) and the ED (4.9 hours), which was longer than the 90^th^ percentile for a wait time of 2.7 hours published for low-acuity patients who were discharged from Ontario EDs.^19^ Longer wait times at the EDs may also have contributed to the high rate of leaving prior to assessment (24.1%), which is strikingly higher than the provincial average of 3%,^19^ despite excluding all these patients from wait-time calculations.

Although some consider the ED and UCC as an option of last resort,^3^,^20^ more than half of our patients reported having family doctors which suggests otherwise. Choice of the ED or UCC for refilling their prescriptions may instead be an ‘affirmative choice’^21^,^22^ driven by a failure to receive adequate help at other sources of care.^9^ Factors that lead patients to seek other sources of care outside of their family doctor include difficulty with accessing complicated appointment systems, English as an additional language, difficulty navigating the telephone, health literacy, and convenience.^18^,^22^,^23^ Our city has not been considered underserviced by family physicians. Of all participants, 15.3% had greater than one visit during the study timeframe with one patient presenting 26 times. It has been suggested that social and economic forces have strong impact on patients who are frequent utilizers of acute care resources for non-urgent problems.^3^

Emergency physicians practicing in this community have reason to be wary about misdirected prescriptions with 49.5% of patients requesting at least one prescription with street value. Relative to population size, it has been estimated that London has one of the largest populations of injection drug users in Canada.^24^ Access to patient drug profiles has become more readily available to emergency physicians, but it is unclear how this affects their prescribing patterns.^5^,^25^

It is important to pay equal attention to the other 50.5% of patients who requested medications without street value. Stopping many of these medications such as insulin, anticoagulants, and anticonvulsants could result in significant adverse health consequences. Psychiatric medications such as antidepressants, antipsychotics, and bipolar agents make up 20% of the total prescriptions requested in a city that has more psychiatrists per capita than the average in Ontario^26^ and no shortage of family physicians.

With such a large number of requests for medications lacking street value, a shift in the accepted boundaries of emergency physician practice may need to be considered. The ‘just say no’ policy of the ED in this study directs physicians away from writing prescriptions for the purpose of continuing care but does allow for exceptions at the discretion of the physician. Intended to protect against medication misdirection, this policy may not be an appropriate response to requests for some of these medications. Our results confirmed that it takes significantly longer to say ‘no’ than to say ‘yes’ to a prescription request but physicians did say ‘no’ to almost 80% of patients.

This time pressure adds yet another tension for physicians and their departments in the era of scorecards that track and reward throughput.^6^ The 90^th^ percentile for LOS was 3.2 hours at the UCC and 5.6 hours at the EDs compared with a provincial report of 3.9 hours for low-acuity patients who were discharged.^19^ Prolonged LOS is of concern to healthcare administrators because of the perceived negative association with cost and crowding.^27^ Some argue that the true cost of serving non-urgent patients is lower than widely believed because of high, fixed operating costs and relatively low marginal costs.^28^,^29^ Our PRPs spent little time actively consuming department resources (medians of 17 and 20 minutes for UCC and ED), which is consistent with the literature.^11^ If these patients neither increase costs to the healthcare system nor contribute significantly to crowding, then the issue of their diversion to another place of care loses much of its relevance.^8^,^11^

Diversion also presumes that there is a primary care system ready and waiting to care for these patients, many of whom are vulnerable with challenging medical and social needs.^7^ A city with an adequate number of family physicians per capita does not necessarily translate into availability of care for all patients. Many diversion plans and implementation solutions are based on the assumptions of healthcare planners, whose lives of privilege differ extraordinarily from the lives of those they serve.^7^ Another questionable assumption is that patients are rational consumers and will make ‘better’ and predictable choices if proper education, incentives, and disincentives are provided.^3^ Ultimately within the current Canadian healthcare system, the decision of where to receive care remains with the patient.

Diversion may not be the only answer. Creative, holistic solutions have been described for ED patients that may be adaptable and beneficial for all involved in the care of PRPs. Malone proposed the implementation of an ED ‘slow track’ for high utilization patients where clinicians work alongside social workers to identify those at risk and address their social, economic, and structural barriers.^3^ This concept may slow throughput for a particular visit but may be beneficial for both the patient and the department in the future. A more recent study from Utah demonstrated the ability to systematically screen and refer for ED patients’ unmet social needs by using existing resources and to link screening results, service referral details, and health service data.^13^

Our study marks a first step in understanding the ‘who’, ‘what’ and ‘where’ of PRPs. Future research is needed to explore the important questions of ‘why’ raised by this study. Why did the patient choose an acute care setting v. their family doctor or another choice? Why did some patients continue to pay return visits: Were they successful in obtaining what they wanted or were they not? Why did physicians decide to offer or decline to write a prescription? Qualitative studies using more interpretative methodologies could delve deeper into these questions, adding important perspective needed to create care strategies.

## LIMITATIONS

There are several limitations to our study. Our reporting of requests for opioid analgesia was falsely low. When a request for ‘pain meds’ was recorded in the triage note with no more specific descriptor, we counted this request as a non-opioid analgesic to avoid overestimating the narcotic request. Future studies collecting data prospectively could remove this limitation. For the period of this study, our electronic health record was a hybrid, with physician notes recorded on a paper chart and all other data recorded electronically. We did not review the paper charts, which may have led to some inaccuracies. We accepted the patient’s report of having a family physician as accurate. Yet there may have been multiple reasons for patient misrepresentation including a perceived improvement in their chances of obtaining a desired prescription. This was supported by the markedly inconsistent documentation of family physicians on review of patients seeking prescriptions on multiple occasions.

This study was undertaken in a medium-sized urban community with a large opioid problem, adequately serviced by family physicians and psychiatrists. Our results signal some of the gaps in healthcare that existed locally at the time of this study, but generalizability to other sites and times may be limited. However, the information gathered should be easily retrievable from most electronic health records and could serve to highlight areas of concern within other communities. Finally, this study took place in Canada, where we have a publicly funded healthcare system, which may also affect generalizability.

## CONCLUSION

Our study is a first step in understanding patients who present to acute care settings for the sole purpose of a prescription refill request. Patients who requested medications of street value and those who did not presented in equal numbers, which would suggest that any ‘one-size-fits-all’ care strategy is inadequate. The time may have arrived for EDs and urgent care centres to expand their approach and become more creative in meeting the needs of these patients.

## Figures and Tables

**Table 1:** Patient and response variables.

Variable	All patients requesting prescriptions N(%)	Patients requesting at least one medication with street value N (%)
Age in years	n = 1,696	n = 740
18 – 30	390 (23.0)	163 (22.0)
31–50	741 (43.7)	355 (48.0)
> 50	565 (33.3)	222 (30.0)
Gender	n = 1,696	n = 740
Male	982 (57.9)	444 (60.0)
Female	708 (41.7)	292 (39.5)
Other	6 (0.4)	4 (0.5)
Family doctor documented*	n = 1694	n = 738
Yes	939 (55.4)	417 (56.5)
No	755 (44.6)	321 (43.5)
Site Visited	n = 1696	n = 740
ED	655 (38.6)	293 (39.6)
UCC	1,041 (61.4)	447 (60.4)
Left without being seen		
ED	n = 655 158 (24.1)	n = 293 58 (19.8)
UCC	n = 1,041 85 (8.1)	n = 447 39 (8.7)
Patients with repeat visits by site	n=173	n=124
ED only	38 (22.0)	27 (21.8)
UCC only	80 (46.2)	57 (46.0)
Both sites	55 (31.8)	40 (32.2)

* Two patients with family Doctor field not completed in record.

*ED*, emergency department; *UCC*, urgent care centre.

**Table 2:** Prescriptions requested and written by category for all patients and patients with repeat visit.

Prescription category	Prescriptions requested by all patients N (%)	Prescriptions written by physician N (%)	Prescriptions requested by patients with repeat visits N (%)
Analgesics-opioid**	481 (21.24)	61 (9.92)	154 (19.47)
Anxiolytics-benzodiazepine**	266 (11.74)	65 (10.57)	130 (16.43)
Analgesics-non opioid	248 (10.95)	50 (8.13)	91 (11.50)
Antidepressants	232 (10.24)	90 (14.63)	68 (8.60)
Antipsychotics	201 (8.87)	53 (8.62)	82 (10.37)
Central nervous system agents-amphetamines**	126 (5.56)	32 (5.20)	68 (8.60)
Cardiovascular agents	124 (5.47)	73 (11.87)	22 (2.78)
Respiratory tract agents	91 (4.02)	45 (7.32)	22 (2.78)
Antibacterials	70 (3.09)	2 (0.33)	12 (1.52)
Gastrointestinal agents	63 (2.78)	26 (4.23)	26 (3.29)
Anticonvulsants	56 (2.47)	17 (2.76)	20 (2.53)
Blood glucose regulators	46 (2.03)	19 (3.09)	10 (1.26)
Blood modifiers-anticoagulants	45 (1.99)	9 (1.46)	14 (1.77)
Sleep disorder agents	35 (1.55)	16 (2.60)	16 (2.02)
Immunological agents	30 (1.32)	4 (0.65)	8 (1.01)
Antivirals	22 (0.97)	6 (0.98)	0 (0.00)
Bipolar agents	18 (0.79)	7 (1.14)	9 (1.14)
Hormonal agents	16 (0.71)	6 (0.98)	5 (0.63)
Contraceptives	14 (0.62)	4 (0.65)	3 (0.38)
Skeletal muscle relaxants	14 (0.62)	7 (1.14)	6 (0.76)
Cannabinoids**	13 (0.57)	4 (0.65)	10 (1.26)
Antiparkinson agents	12 (0.53)	2 (0.33)	7 (0.88)
Antiemetics	8 (0.35)	1 (0.16)	3 (0.38)
Electrolytes/minerals/metals/vitamins	8 (0.35)	1 (0.16)	4 (0.51)
Anti-addiction/substance abuse treatment agents	5 (0.22)	2 (0.33)	0 (0.00)
Ophthalmic agents	4 (0.18)	5 (0.81)	0 (0.00)
Genitourinary agents	4 (0.18)	2 (0.33)	0 (0.00)
Antimigraine agents	3 (0.13)	0 (0.00)	1 (0.13)
Sexual disorder agents	3 (0.13)	3 (0.49)	0 (0.00)
Antifungals	2 (0.09)	0 (0.00)	0 (0.00)
Antiparasitics	2 (0.09)	0 (0.00)	0 (0.00)
Metabolic bone disease agents	2 (0.09)	0 (0.00)	0 (0.00)
Dermatological agents	1 (0.04)	3 (0.49)	0 (0.00)
	n = 2,265	n = 615	n = 791

Note: The requested prescriptions were categorized using the United States Pharmacopeial Convention Drug Classification System^17^ with the exception of cannabinoids, which was added to reflect the Canadian content.

** Indicates categories with street value.

## References

[1] Derlet RW, Kinser D, Ray L (1995). Prospective identification and triage of nonemergency patients out of an emergency department: a 5-year study. Ann Emerg Med.

[2] LaCalle E, Rabin E (2010). Frequent users of emergency departments: the myths, the data, and the policy implications. Ann Emerg Med.

[3] Malone RE (1998). Whither the almshouse? Overutilization and the role of the emergency department. J Health Polit Policy Law.

[4] Grover CA, Close RJ, Wiele ED (2012). Quantifying drug-seeking behavior: a case control study. J Emerg Med.

[5] Weiner SG, Griggs CA, Mitchell PM (2013). Clinician impression versus prescription drug monitoring program criteria in the assessment of drug-seeking behavior in the emergency department. Ann Emerg Med.

[6] Browne AJ, Smye VL, Rodney P (2011). Access to primary care from the perspective of Aboriginal patients at an urban emergency department. Qual Health Res.

[7] Koziol-McLain J, Price DW, Weiss B (2000). Seeking care for nonurgent medical conditions in the emergency department: through the eyes of the patient. J Emerg Nurs.

[8] Afilalo J, Marinovich A, Afilalo M (2004). Nonurgent emergency department patient characteristics and barriers to primary care. Acad Emerg Med.

[9] Han A, Ospina MB, Blitz S (2007). Patients presenting to the emergency department: the use of other health care services and reasons for presentation. CJEM.

[10] Gill JM, Mainous AG, Nsereko M (2000). The effect of continuity of care on emergency department use. Arch Fam Med.

[11] Schull MJ, Kiss A, Szalai JP (2007). The effect of low-complexity patients on emergency department waiting times. Ann Emerg Med.

[12] Lowe RA, Schull M (2011). On easy solutions. Ann Emerg Med.

[13] Wallace AS (2020). Implementing a social determinants screening and referral infrastructure during routine emergency department visits, Utah, 2017–2018. Prev Chronic Dis.

[14] Worster A, Bledsoe RD, Cleve P (2005). Reassessing the methods of medical record review studies in emergency medicine research. Ann Emerg Med.

[15] Canadian Institute for Health Information TCEDISC (2015). The Canadian Emergency Department Diagnosis Shortlist.

[16] Elm E, Altman DG, Egger M (2008). The Strengthening the Reporting of Observational Studies in Epidemiology (STROBE) Statement: guidelines for reporting observational studies. J Clin Epidemiol.

[17] Safety HQ (2020). USP DC (Drug Classifications).

[18] Schumacher JR, Hall AG, Davis TC (2013). Potentially preventable use of emergency services: the role of low health literacy. Med Care.

[19] Ontario HQ (2016). Under pressure: emergency department performance in Ontario.

[20] Iserson KV, Kastre TY (1996). Are emergency departments really a “safety net” for the medically indigent?. Am J Emerg Med.

[21] Bernstein SL (2006). Frequent emergency department visitors: the end of inappropriateness. Ann Emerg Med.

[22] Ragin DF, Hwang U, Cydulka RK (2005). Reasons for using the emergency department: results of the EMPATH Study. Acad Emerg Med.

[23] MacKichan F, Brangan E, Wye L (2017). Why do patients seek primary medical care in emergency departments? An ethnographic exploration of access to general practice. BMJ Open.

[24] Middlesex-London Health Unit (2017). Development and implementation of a strategy to address HIV, hepatitis C, invasive group A streptococcal disease and infective endocarditis in persons who inject drugs in Middlesex-London, Ontario (Report No. 021-17).

[25] Grover CA, Garmel GM (2012). How do emergency physicians interpret prescription narcotic history when assessing patients presenting to the emergency department with pain?. Perm J.

[26] Kurdyak P, Zaheer J, Cheng J (2017). Changes in characteristics and practice patterns of Ontario psychiatrists: implications for access to psychiatrists. Can J Psychiatry.

[27] Durand AC, Gentile S, Devictor B (2011). ED patients: how nonurgent are they? Systematic review of the emergency medicine literature. Am J Emerg Med.

[28] Williams RM (1996). The costs of visits to emergency departments. N Eng J Med.

[29] Richardson LD, Hwang U (2001). Access to care a review of the emergency medicine literature. Acad Emerg Med.

